# Astragalus polysaccharides improve cardiomyopathy in STZ-induced diabetic mice and heterozygous (SOD2+/-) knockout mice

**DOI:** 10.1590/1414-431X20176204

**Published:** 2017-07-10

**Authors:** J. Ju, W. Chen, Y. Lai, L. Wang, H. Wang, W.J. Chen, X. Zhao, H. Ye, Y. LI, Y. Zhang

**Affiliations:** 1 Department of Geriatrics, Huashan Hospital, Fudan University, Shanghai, China; 2 Department of Endocrinology, Huashan Hospital, Fudan University, Shanghai, China; 3 Core Center of Clinical Skill Training, Shanghai Medical College, Fudan University, Shanghai, China; 4 Experimental Center of Basic Medicine, Shanghai Medical College, Fudan University, Shanghai, China

**Keywords:** Diabetes, Cardiomyopathy, Oxidative stress, Superoxide dismutase

## Abstract

Oxidative stress plays an important role in the development of diabetic cardiomyopathy. In the present study, we determined whether the effect of astragalus polysaccharides (APS) on diabetic cardiomyopathy was associated with its impact on oxidative stress. Streptozotocin (STZ)-induced diabetic mice and heterozygous superoxide dismutase (SOD2+/-) knockout mice were administered APS. The hemodynamics, cardiac ultrastructure, and the apoptosis, necrosis and proliferation of cardiomyocytes were assessed to evaluate the effect of APS on diabetic and oxidative cardiomyopathy. Furthermore, H_2_O_2_ formation, oxidative stress/damage, and SOD activity in cardiomyocytes were evaluated to determine the effects of APS on cardiac oxidative stress. APS therapy improved hemodynamics and myocardial ultrastructure with reduced apoptosis/necrosis, and enhanced proliferation in cardiomyocytes from both STZ-induced diabetic mice and heterozygous SOD2+/- knockout mice. In addition, APS therapy reduced H_2_O_2_ formation and oxidative stress/damage, and enhanced SOD activity in both groups of mice. Our findings suggest that APS had benefits in diabetic cardiomyopathy, which may be partly associated with its impact on cardiac oxidative stress.

## Introduction

Cardiomyopathy in diabetes can develop independently from elevated blood pressure or coronary artery disease, which is termed as “diabetic cardiomyopathy (DCM)”. This is characterized by the function and structure of the myocardium, including ventricular dilation, myocardial fibrosis and cardiac dysfunction (1). The idea that oxidative stress contributes to DCM is widely acknowledged. The activation of oxidative stress, increased production of mitochondrial reactive oxygen species (ROS) and the subsequent mitochondrial pathway of apoptosis by hyperglycemia are regarded as the major causes of clinical cardiomyopathy associated with diabetes (2,3). Superoxide dismutase (SOD) is an essential enzyme that catalyzes highly reactive superoxide into hydrogen peroxide. Due to its localization within the mitochondria, manganese SOD (Mn-SOD, coded by the *SOD2* gene) is the most effective member of the SOD family of enzymes in protecting the mitochondria from oxidative stress induced by enzymatically scavenging superoxide anions (4,5). Heterozygous (SOD2+/-) knockout mice have a 50% reduction in SOD2 activity with increased mitochondrial oxidative damage, leading to reduced myocardial antioxidant defenses, and causing enlarged/dilated hearts and severe cardiomyopathy. Hence, this has thereby been utilized to explore oxidative stress in DCM (6–8).

Astragalus (the root of *Astragalus membranaceus*, *huang qi*) is a traditional Chinese herbal medicine that has been widely used in the clinical therapy of heart failure (9). Astragalus polysaccharides (APS) are hydrosoluble components, and are the main bioactive ingredient extracted from Astragalus. Previously, it was found that APS therapy could rescue heart dysfunction in both streptozotocin (STZ)-induced diabetic mice and db/db mice (9–14). However, it remains unclear whether the beneficial effect of APS on DCM is associated with its impact on cardiac oxidative stress. Therefore, STZ-induced diabetic mice and heterozygous SOD2+/- knockout mice administered with APS were employed in the present study, and cardiac function, cardiac apoptosis/necrosis and cardiac oxidative damage were evaluated to explore the potential mechanism.

## Material and Methods

### Animals and treatments

This study protocol was approved by the Institutional Animal Care and Use Committee of Shanghai Fudan University Medical Center, Shanghai, China, and conforms to the guidelines of the National Research Council for Laboratory Animal Care in Research.

Male heterozygous SOD2-deficient mice (SOD2+/-, with the C57BL/6J background) and C57BJ/6J control mice were used in this study (Jackson Laboratories, USA). Diabetes was induced *via* intraperitoneal injection of STZ, at 50 mg/kg body weight per day for 5 days (Sigma Chemical Co., USA) at 6-week-old mice. Hyperglycemic mice with blood glucose >15 mmol/L were considered diabetic. After diabetes mellitus (DM) induction, mice were administered, or not, APS (2000 mg/kg body weight per day) by gavage for 16 weeks. APS was obtained from Shanghai Institute of Physiology Academia Sinica, China. Non-diabetic age-matched SOD2+/- mice received the same treatment protocol. All mice were housed in the Animal Facility of Shanghai Fudan University Medical Center. Glucose blood levels (monitored by Accu-Check, Roche, USA) were measured weekly. At 24 weeks of age, mice were anesthetized with ketamine chloride at 40 mg/kg of body weight by intraperitoneal injection (*ip*; Imalgene, Italy) plus medetomidine hydrochloride at 0.15 mg/kg of body weight (*ip*; Domitor, Pfizer, Italia S.r.l., Latina, Italy). A hemodynamic study was performed, and then mice were sacrificed by cervical dislocation, and the hearts were harvested for further experiments.

### Hemodynamic study

The right carotid artery was cannulated with a 20 GA 1.1×48 mm Angio-catheter (BD Bioscience Pharmigen, Italy) to record arterial pressure. Then, the aortic catheter was advanced into the left ventricle to measure the left ventricular systolic pressure (LVSP), left ventricular end-diastolic pressure (LVEDP), and the maximal rise/fall rates in left ventricle pressure development (+/-dP/dt_max_). All pressure data were recorded with a MedLab data acquisition system (Shanghai Med Ease Co., China).

### Ultrastructural evaluations

Heart samples were evaluated using an electron microscope to define the ultrastructural changes. Briefly, samples were cut, fixed in diluted Karnovsky's fixative, and stained with uranyl acetate and lead citrate. Then, the samples were viewed on a Philips Morgagni electron microscope (Philips, Netherlands).

### Isolation of myocytes

After the mice were sacrificed, the hearts were harvested, and myocytes from the left ventricle were enzymatically isolated. Briefly, retrograde perfusion was performed on hearts through the aorta with a modified commercial minimal essential medium Eagle Joklik (HEPES-MEM, Worthington Biochemical Corp., USA). The collagenase perfusion (type I, Worthington Biochemical Corp.) of the hearts was performed at 37°C using a HEPES-MEM gassed with 15% N_2_ and 85% O_2_. The left ventricle was minced into small pieces, weighted and shaken in re-suspension medium at 37°C. Then, the ventricular cardiomyocytes were enriched by centrifugation and harvested.

### Detection of apoptosis, necrosis and proliferation

The cell death of cardiomyocytes was measured by *in situ* ligation of hairpin oligonucleotide probes, and myocyte proliferation was determined by immunohistochemistry. Briefly, the sections of the ventricle were treated with protease K and incubated with biotinylated hairpin probe with a single base 3′overhang (hairpin 1) or hairpin oligonucleotide probe with blunt end (hairpin 2) probes (both from Synthetic Genetics) in the presence of T4 DNA ligase. Ligated oligonucleotides were detected with FITC-avidin. Hairpin 1 was utilized to detect for double-stranded DNA breaks in apoptotic cells, while hairpin 2 counted for a form of DNA damage present in nuclei of cells undergoing necrosis. Nuclear marker Ki67 antibodies (BD Bioscience Pharmigen) were employed to identify proliferating cells, and this assessment was performed using a Bio-Rad Radiance 2100 MP (USA) multiphoton microscope and the ImagePro analysis software.

### Total SOD enzyme activity assay

Protein concentrations were measured using a BCA protein kit (Bio-RAD). SOD activity in myocytes was determined using a Superoxide Dismutase Assay Kit (Trevigen, USA), according to manufacturer's instructions. The reaction involved xanthine and xanthine oxidase converting nitroblue tetrazolium (NBT) to NBT-diformazan, generating superoxide radicals, followed by SOD forming hydrogen peroxide (H_2_O_2_) from superoxide radicals. Total SOD activity was determined by the extent of reduction in the appearance of NBT-diformazan.

### Detection of H_2_O_2_, oxidative stress and oxidative damage

H_2_O_2_ production in myocytes were measured using the fluorescent dye 5-(6)-chloromethyl-2′,7′-dichlorodihydrofluoresein diacetate (CM-H_2_DCFDA; Invitrogen, Molecular Probes). Briefly, cells were loaded with CM-H_2_DCFDA for 30 min. The signal generated by CM-H_2_DCFDA was directly proportional to the intracellular H_2_O_2_ concentration. Nuclei were stained by Syto17, which were capable of entering living cells and binding to the DNA. The generation of fluorescence calibration curves and the evaluation of cell brightness were measured using InSpeck Microscopy Image Intensity Calibration microspheres (Molecular Probes), and H_2_O_2_ formation were measured using Bio-Rad Radiance 2100 MP multiphoton microscope and ImagePro analysis software (Media Cybernetics, USA).

Oxidative damage in myocytes was determined as follows. Nitrotyrosine antibody (from Upstate, USA) was employed to detect the oxidative damage to cytoplasmic proteins in myocytes. The 8-OH-deoxyguanosine (8-OH-dG) mouse monoclonal antibody (QED Bioscience, USA) was utilized to determine the oxidative stress in the nuclei in myocytes. The measurement was performed using Bio-Rad Radiance 2100 MP multiphoton microscope and the ImagePro analysis software.

### Statistical analysis

Results are reported as means±SE from 7 replicates. Data was analyzed by one-way ANOVA and *post hoc* tests with GraphPad Prism 5 (GraphPad, USA). P<0.05 was considered to be statistically significant.

## Results

### Protection of cardiac function by APS in diabetic and SOD2+/- hearts

Our previous report suggested that APS treatment ameliorated cardiac dysfunction and protected cardiac function in diabetic mice (9–14). In the present study, it was found that not only STZ-treated mice, but also SOD2+/- mice exhibited deteriorated cardiac phenotypes, including a decrease in LVSP and LV +/-dP/dt, together with an increase in LVEDP. However, after APS treatment, the hemodynamic disorder in both diabetic and SOD2+/- mice was significantly modified, which was comparable to the extent of that in C57BJ/6J control mice. Moreover, the abnormalities in ventricular function and myocardial loading in diabetic SOD2+/- mice, including the decrease in LVSP, +dP/dt and -dP/dt, in combination with the increase in LVEDP, were all significantly reversed after APS administration (Table 1). Thus, the findings in this study suggested that APS treatment could mainly prevent and delay defects in cardiac diastolic and systolic function with oxidative stress and/or diabetes.

**Table 1.** Astragalus polysaccharides (APS) rescued cardiac dysfunction in diabetic mice at 24 weeks.

	Ctrl	DM	DM-APS	SOD2+/-	APS-SOD2+/-	DM-SOD2+/-	APS-DM-SOD2+/-
Heart rate (bpm)	506±16	516±14	502±12	520±16	508±11	522±12	500±13
LV systolic pressure (mmHg)	98.1±2.1	82.5±3.0+	97.2±2.2*	83.6±2.3+	98.6±1.8#	81.5±3.6+	96.8±1.2&
LVPW (mm)	1.11±0.06	1.42±0.03+	1.11±0.08*	1.44±0.07+	1.12±0.06#	1.45±0.08+	1.11±0.03&
IVS (mm)	0.70±0.01	0.84±0.04+	0.72±0.03*	0.85±0.06+	0.72±0.02#	0.86±0.07+	0.73±0.02&
LVID (mm)	3.2±0.02	3.6±0.04+	3.3±0.03*	3.7±0.03+	3.3±0.03#	3.8±0.08+	3.3±0.04&
LVFS (%)	60±2.8	36±1.4+	56±2.4*	34±1.3+	55±2.3#	32±1.0+	52±2.0&
LV end diastolic pressure (mmHg)	7.6±1.2	11.8±3.2+	7.8±1.6*	12.1±2.4+	7.3±1.0#	12.2±1.8+	7.4±0.9&
LV dP/dt_max_ (mmHg/s)	8342±477	5110±602+	8258±352*	5225±424+	8224±245#	5028±612+	8210±312&
LV dP/dt_min_ (-mmHg/s)	6840±573	4442±325+	6702±477*	4612±288+	6460±498#	4218±250+	6512±388&

Data are reported as means±SE. DM: diabetic mice; DM-SOD2+/-: diabetic SOD2+/- mice; APS-DM-SOD2+/-: diabetic SOD2+/- mice with APS treatment; APS-DM: diabetic mice with APS treatment (n=20 per group); LV: left ventricle; LVPW: left ventricular posterior wall thickness; IVS: inter-ventricular septal wall thickness; LVID: left ventricular internal diameter; LVFS: left ventricular fractional shortening.

* P<0.05 *vs* diabetic mice;

# P<0.05 *vs* SOD2+/-mice;

& P<0.05 *vs* diabetic SOD2+/- mice;

+ P<0.05 *vs* C57BJ/6J control mice.

### Prevention of ultrastructural abnormalities by APS in diabetic and SOD2+/- hearts

**Figure 1.**
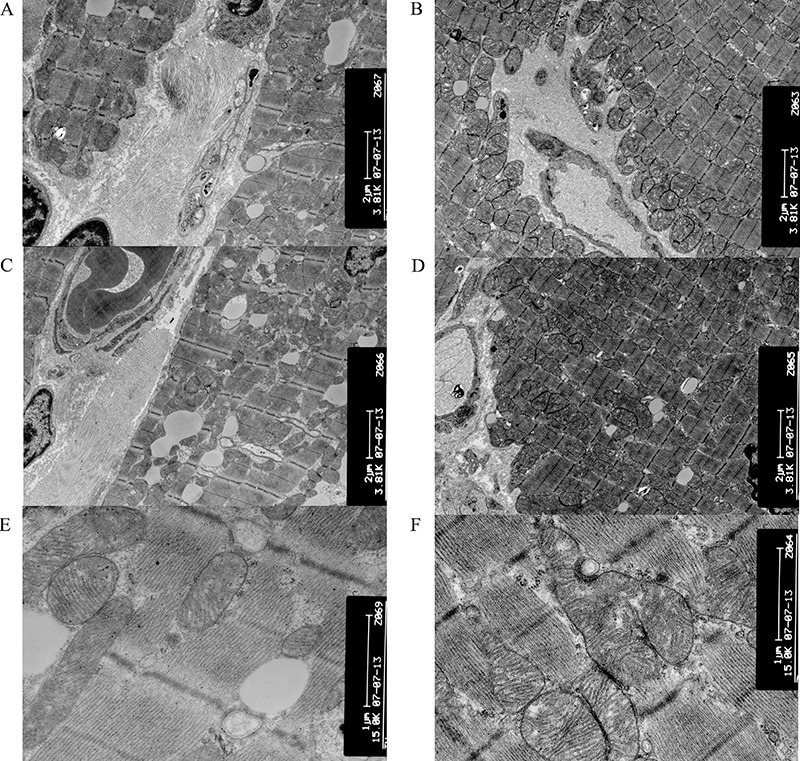
Astragalus polysaccharides (APS) ameliorated ultrastructural abnormalities in diabetic hearts. Heart samples were evaluated using an electron microscope to define cardiac mitochondrial structural changes. Representative ultrastructural profiles of ventricular cardiac myocytes using a transmission electron microscope (original magnification: ×5,700 in A-D, ×23,300 in E and F). *A*, Diabetic mice; *B*, diabetic mice with APS treatment; *C*, superoxide dismutase knockout (SOD2+/-) mice; *D*, SOD2+/- mice with APS treatment; *E*, diabetic SOD2+/- mice; *F*, diabetic SOD2+/- mice with APS treatment.

Electron microscopy examination suggested severe damage in the myocardial ultrastructure of diabetic, SOD2+/- and diabetic SOD2+/- hearts, which included disrupted sarcomeres, tubules and mitochondria, in comparison to that from control hearts. Notably, the disarrangement of the varying size and shape of mitochondria, the disruption of the mitochondrial cristae and increased abundance of peroxisomes were also found in diabetic and/or SOD2+/- hearts. However, the myocardial ultrastructure of diabetic and/or SOD2+/- hearts were well-protected and improved by APS treatment, which was characterized by integrated mitochondria, regular sarcomeres, intact endoplasmic reticulum, and undisrupted chondriosome. Moreover, APS-treated diabetic and/or SOD2+/- myocardium exhibited parallel arrangements and practically normal shapes of mitochondria with integrated mitochondrial membrane and cristae, and less abundance of peroxisomes (Figure 1). The results of the present study revealed that ultrastructural pathological changes including those presented in diabetic hearts with or without partial SOD2 depletion could be attenuated and prevented by APS treatment.

### Effects of APS on apoptosis, necrosis and proliferation in diabetic and SOD2+/- hearts

**Figure 2.**
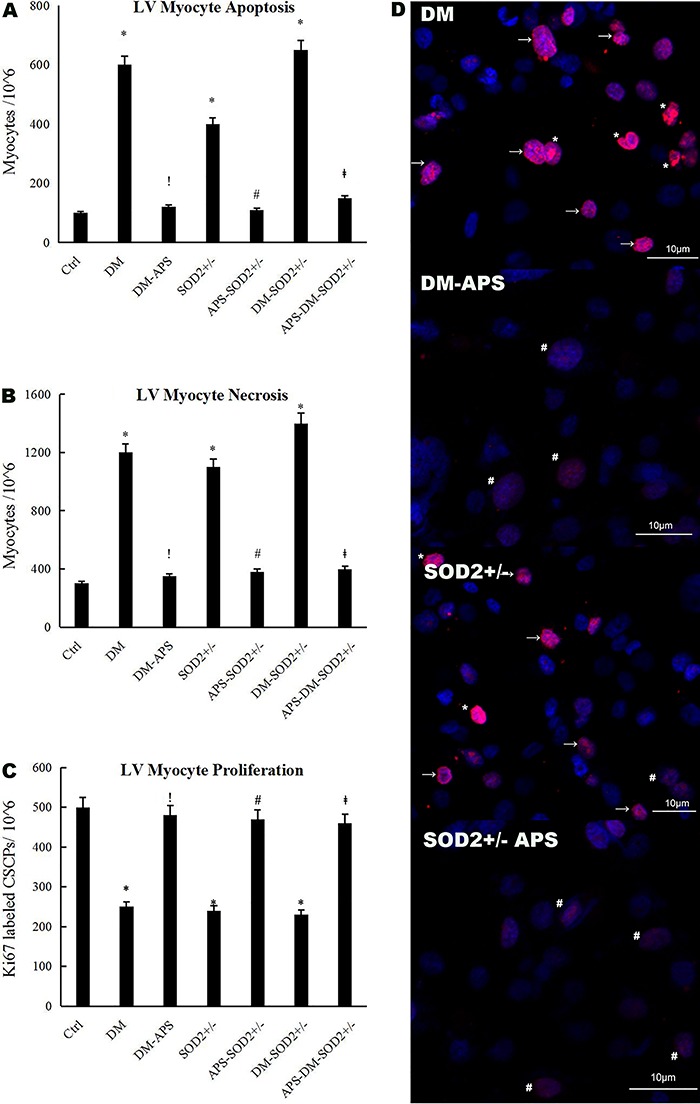
Astragalus polysaccharides (APS) affected the apoptosis, necrosis and proliferation of the myocardium in diabetes. *A*, Myocyte apoptosis in the left ventricle (detected by hairpin 1); *B*, myocyte necrosis in the left ventricle (detected by hairpin 1 and hairpin 2); *C*, proliferation of myocytes in the left ventricle. Data are reported as means±SE. ^!^P<0.05 *vs* diabetic mice (DM); ^#^P<0.05 *vs* SOD2+/-mice; ^?^P<0.05 *vs* diabetic SOD2+/- mice; *P<0.05 *vs* C57BJ/6J control mice (one-way ANOVA). *D*, Immunohistochemistry microphotographs showing the apoptosis, necrosis and proliferation of cardiomyocytes derived from the left ventricle (white arrows: apoptosis; white asterisk: necrosis; #: proliferation). LV: left ventricle.

Our data revealed that apoptosis in the left ventricle from diabetic mice, SOD2+/- mice and diabetic SOD2+/- mice increased to almost 10-, 9-, and 10.5-fold, respectively, in comparison with that from C57BJ/6J control mice. However, the increase in myocyte apoptosis were all significantly reversed by APS treatment in diabetic mice, SOD2+/- mice and diabetic SOD2+/- mice (Figure 2A). Furthermore, diabetes and/or oxidative stress by partial SOD2 depletion increased myocytes necrosis by 4- to 6-fold in diabetic mice, SOD2+/- mice and diabetic SOD2+/- mice, compared with that from C57BJ/6J control mice. In addition, APS treatment also reversed the increase in myocyte necrosis of the left ventricle with diabetes and/or partial SOD2 depletion (Figure 2B). The results of the present study suggest that APS might have a beneficial effect on the apoptosis and necrosis of cardiomyocytes with either diabetes and/or oxidative stress by partial SOD2 depletion.

Subsequently, Ki67 antibodies were employed in the left ventricle to identify cycling cells. As shown in the present study, the percentage of Ki67-labeled myocytes in the left ventricle, with diabetes and/or partial SOD2 depletion, all decreased by 50%, compared with that in C57BJ/6J control hearts, while the decrease in myocyte proliferation in hearts with diabetes and/or partial SOD2 depletion was significantly inhibited by APS treatment (Figure 2C). These results raised the possibility that APS treatment might affect myocyte division and enhance myocyte proliferation in cardiomyocytes with diabetes and/or partial SOD2 depletion.

### Effect of APS on SOD activities in diabetic and SOD2+/- hearts

**Figure 3.**
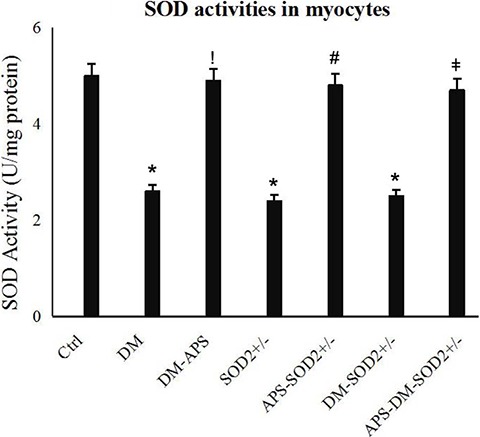
Astragalus polysaccharides (APS)-enhanced superoxide dismutase (SOD) activity of the myocardium in diabetes. Data are reported as mean±SE. ^!^P<0.05 *vs* diabetic mice (DM); ^#^P<0.05 *vs* SOD2+/- mice; ^?^P<0.05 *vs* diabetic SOD2+/- mice; *P<0.05 *vs* C57BJ/6J control mice (one-way ANOVA).

In the present study, it was found that diabetes inhibited SOD activity in control hearts to the extent of the inhibition of SOD activity in hearts by partial SOD2 depletion alone, suggesting that diabetes had a negative effect on SOD activity in myocytes (Figure 3). After APS administration, SOD activities in both diabetic and SOD2+/- myocytes were significantly elevated to the extent of the levels in myocytes from healthy controls. In addition, APS treatment markedly reversed the reduction of SOD activity in myocytes in diabetic SOD2+/- mice, compared with that in C57BJ/6J control mice (Figure 3). Thus, our findings suggest that APS treatment might have beneficial effects on SOD activity in cardiomyocytes, which were mainly deteriorated by diabetes.

### Reduction of H_2_O_2_ formation and oxidative stress by APS in diabetic and SOD2+/- hearts

**Figure 4.**
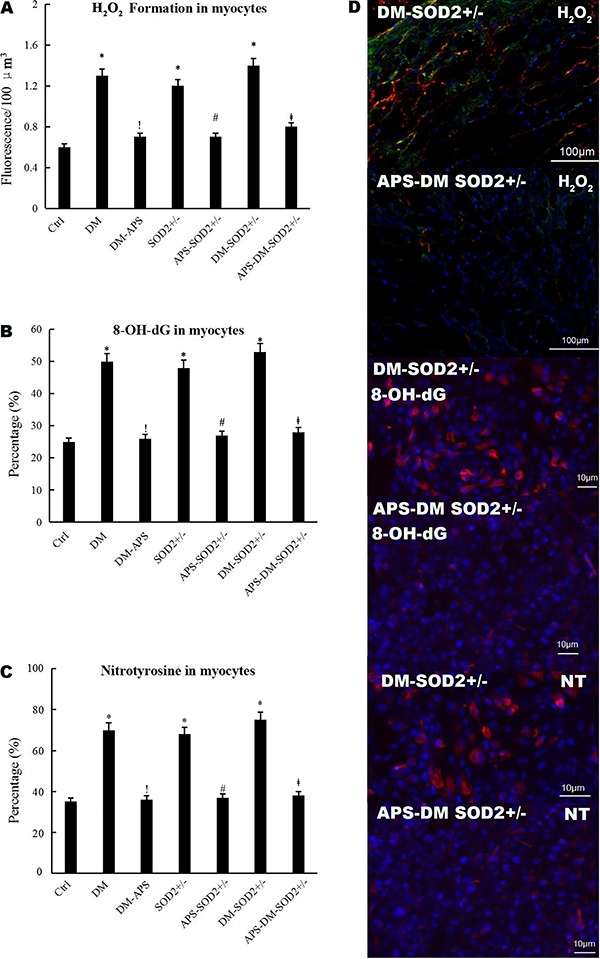
Astragalus polysaccharides (APS) inhibited H_2_O_2_ formation and oxidative stress/damage in diabetic hearts. *A*, H_2_O_2_ formation in myocytes in the left ventricle. *B*, Oxidative stress in the nuclei in the left ventricle. *C*, Oxidative damage to cytoplasmic proteins in the left ventricle. Data are reported as means±SE. ^!^P<0.05 *vs* diabetic mice (DM); ^#^P<0.05 *vs* SOD2+/- mice; ^?^P<0.05 *vs* diabetic SOD+/- mice; *P<0.05 *vs* C57BJ/6J control mice (one-way ANOVA). *D*, Fluorescence microphotographs showing H_2_O_2_ formation of cardiomyocytes (green: fluorescence for H_2_O_2_; blue: cardiomyocytes), and immunohistochemistry microphotographs showing oxidative stress of cardiomyocytes derived from the left ventricle (red: immunohistochemistry for NT or 8-OH-dG; blue: cardiomyocytes).

The intracellular levels of H_2_O_2_ were measured to evaluate part of the ROS formation in myocytes, which were loaded with CM-H_2_DCFDA. Compared with that in C57BJ/6J control hearts, the generation of H_2_O_2_ nearly doubled in myocytes from hearts with diabetes and/or with partial SOD2 depletion. After APS treatment, H_2_O_2_ formation did not significantly increase in myocytes from hearts with diabetes and/or with partial SOD2 depletion (Figure 4A). Thus, these data suggest that H_2_O_2_ formation in cardiomyocytes enhanced by diabetes and partial SOD2 depletion could partly be attenuated by APS treatment.

In order to explore ROS-mediated cytoplasmic and DNA-damage, nitrotyrosine and 8-OH-deoxyguanosine were respectively employed in myocytes. Our data revealed that the percentages of both nitrotyrosine-labeled myocytes and 8-OH-dG labeled myocytes increased by 3-fold to 4-fold in hearts with diabetes and/or with partial SOD2 depletion, compared with that in C57BJ/6J control hearts. This increase was significantly reversed by APS treatment in hearts with diabetes and/or with partial SOD2 depletion (Figure 4B and C). These results suggest that cardiac oxidative stress could be partly reduced by APS therapy in hearts with partial SOD2 depletion in the absence or presence of diabetes.

## Discussion

DCM consists of a series of structural and functional changes, including chronic loss of myocytes and vascular cells, leading to decreased muscle mass, chamber dilation, impaired systolic and diastolic ventricular function, and finally an association with left ventricular dysfunction, which is directly associated with hyperglycemia and independently from elevated blood pressure or coronary artery disease (2,3). Our findings in the present study suggest that APS administration could positively improve ventricular function and hemodynamics in STZ-induced diabetic hearts, together with the wall protection of the myocardial ultrastructure. Moreover, APS therapy could remarkably reduce the apoptosis and necrosis of cardiomyocytes from STZ-induced diabetic hearts, and at the same time, enhance the cardiac proliferation of STZ-induced diabetic hearts. These findings indicate that APS therapy might have beneficial effects on DCM.

Emerging data from experimental, pathological and clinical studies have demonstrated that increased oxidative stress or the overproduction of H_2_O_2_ (part of ROS) by the mitochondria is the central and major role contributing to DCM (15,16). The increase in ROS serves to decrease the antioxidant capacity of the diabetic myocardium, contributing significantly to apoptotic cell death, which finally is associated with cardiac morphological and functional abnormalities, causing the onset or development DCM (17,18). In the present study, the generation of H_2_O_2,_ oxidation-mediated cytoplasmic and DNA damage in cardiomyocytes from STZ-induced diabetic mice all markedly increased compared with those from normal control mice. This suggests that oxidative stress might play an important role in STZ-diabetic cardiomyopathy. However, our further findings revealed that APS administration remarkably reduced the oxidative damage to cytoplasmic proteins in cardiomyocytes from STZ-induced diabetic mice, and the oxidative stress in the nuclei in cardiomyocytes. In addition, the cardiac generation of H_2_O_2_ from STZ-induced diabetic hearts was reversed by APS therapy. These findings suggest that the positive effect of APS therapy on diabetic cardiomyopathy might be partly associated with its impact of cardiac oxidative stress.

As the primary antioxidant system, the SOD family is highly conserved, with most species having a cytoplasmic SOD (SOD1), a mitochondrial SOD (SOD2), and an extracellular SOD (SOD3) (19). Mitochondria are a major intracellular source of oxidative stress, and the superoxide anion radical exerts its effects locally, and poorly penetrates the membranes. Therefore, in mitochondria, SOD2 is the crucial and major scavenger for superoxide. Superoxide is covered by SOD2 to H_2_O_2,_ and the reduction of H_2_O_2_ is catalyzed by mitochondrial glutathione peroxidase to H_2_O (20). The present study revealed that APS administration mainly enhanced cardiac SOD activity in STZ-induced diabetic mice together with the decrease in cardiac H_2_O_2_ generation, indicating that the action of APS might partly be related with SOD activity. In addition, in heterozygous SOD2+/- knockout mice, APS administration could definitely improve cardiac dysfunction and modify cardiac hemodynamic disorder, including the decrease in LVSP, +dP/dt and-dP/dt, together with the increase in LVEDP, which indicate that APS therapy could protect cardiac function in oxidative cardiomyopathy. In addition, APS administration protected the myocardial ultrastructure, especially the integrity of mitochondria, reduced cell apoptosis and necrosis, and enhanced cell proliferation in heterozygous SOD2+/- knockout hearts, which suggest that APS therapy could protect cardiomyocytes in oxidative cardiomyopathy. Furthermore, APS treatment inhibited cardiac H_2_O_2_ formation, reduced cardiac oxidative stress/damage, and increased SOD activity in cardiomyocytes from heterozygous SOD2+/- knockout hearts with or without diabetes. This indicates that APS therapy could mainly reduce oxidative stress in oxidative cardiomyopathy, and that this might be partly related with SOD activity. However, in our study, the levels of ·OH and O_2_·- and the activities of catalase or glutathione peroxidase were not assessed. Therefore, further experiments should be performed to explore the mechanism proposed by the action of APS.

Taking all these findings together, the present study revealed that APS therapy could improve cardiomyopathy in STZ-induced diabetic mice and heterozygous SOD2+/- knockout mice with reduced cardiac apoptosis/necrosis and enhanced cardiac proliferation, leading to a marked recovery of ventricular function and protection of myocardial ultrastructure. Importantly, APS therapy could reduce cardiac H_2_O_2_ generation and oxidative stress/damage with the increase of SOD activity in cardiomyocytes from STZ-induced diabetic hearts and heterozygous SOD2+/- knockout hearts. Thus, our findings suggest that APS has benefits in diabetic cardiomyopathy, which may be partly associated with its impact on cardiac oxidative stress.
